# Psychosocial determinants of oral health outcomes in young children

**DOI:** 10.3389/fped.2024.1478302

**Published:** 2024-12-06

**Authors:** Dorota T. Kopycka-Kedzierawski, Patricia G. Ragusa, Changyong Feng, Kim Flint, Gene E. Watson, Cynthia L. Wong, Steven R. Gill, Ronald J. Billings, Thomas G. O’Connor

**Affiliations:** ^1^ Department of Oral and Craniofacial Sciences, Eastman Institute for Oral Health, University of Rochester, Rochester, NY, United States; ^2^ Department of Biostatistics and Computational Biology and Department of Anesthesiology and Perioperative Medicine, University of Rochester, Rochester, NY, United States; ^3^ Department of Immunology and Microbiology, University of Rochester, Rochester, NY, United States; ^4^ Departments of Psychiatry, Neuroscience, and Obstetrics and Gynecology, University of Rochester, Rochester, NY, United States

**Keywords:** oral health, early childhood caries, social conditions, social determinants of health, International Caries Detection and Assessment System (ICDAS)

## Abstract

**Objective:**

To examine the social determinants of early childhood caries (ECC), one of the greatest public health risks affecting children, and examine alternative pathways of influence.

**Methods:**

A physically healthy, socio-demographically high-risk sample of initially caries-free children, aged 1–4 years, was prospectively studied for 2 years. At 6-month intervals, assessments were made of caries presence from a standard dental exam; oral microbiology was assayed from saliva samples; oral hygiene behaviors and psychological and psychosocial risk exposure were derived from interviews and questionnaires.

**Results:**

189 children were enrolled; ECC onset occurred in 48 children over the 2-year study period. A composite measure of psychosocial risk was significantly associated with ECC onset over the course of the study (1.57, 95% CI 1.12–2.20, *p* < .001) and significantly associated with multiple risks for ECC, including poor diet/feeding (.92; 95% CI. 22–1.61, *p* < .01), poor oral hygiene (.39; 95% CI .09–.68), *p* < .05), and higher concentrations *Lactobacilli* (.96; 95% CI .43–1.49, *p* < .001). Multivariable regression analyses provided indirect support for the hypothesis that psychosocial risk exposure predicts ECC onset via behavioral and oral hygiene pathways.

**Conclusions:**

The study provides novel evidence that psychosocial factors influence many of the purported risks for ECC and strong evidence that there are social and psychological determinants of ECC onset.

## Introduction

The expansive research literature on the social determinants of health emphasizes that social-psychological risk exposure early in development predicts diverse health outcomes in adulthood. An important extension of this work, because of its implications for understanding disease mechanisms and timing of clinical intervention, is research documenting that early risk exposure also predicts clinical health conditions with *childhood onset*. The current study examines psychosocial determinants of early childhood caries (ECC), one of the most prevalent early-onset clinical and public health concerns which has an onset before age 5 years and holds long-term implications for oral and craniofacial health and well-being (1–4).

Evidence of a socio-economic and socio-demographic gradient of ECC has been widely reported: higher rates of ECC are reliably found among children in low income/low resource families and minoritized communities (5, 6). Although often interpreted to imply a social-psychological etiology (e.g., stress), research findings linking socio-demographic context to ECC are confounded by many alternative mechanisms of risk which also show a social-demographic gradient (7–11) and pose compelling alternative explanations and confounds. More direct evidence regarding the role of specific psychological, social and behavioral risk factors for ECC is needed to inform the type and timing of targeted (preventive) interventions to improve child oral health outcomes. That is the aim of the current study, which was designed to test alternative pathways through which social-psychological factors may predict ECC. Leverage for testing social-psychological determinants of ECC derives from (a) a prospective longitudinal follow-up of initially caries-free children for 2 years; (b) collection of oral microbiology that has been causally linked with ECC; and (c) detailed assessment of diet, oral hygiene, and economic and demographic covariates.

## Material and methods

### Sample

A cohort of confirmed caries-free pre-school children was recruited from a university-based community pediatric dental clinic in the Northeast US; the longitudinal study was conducted between February 2016 and February 2021. Inclusion criteria were: (a) child age of 1–3 years at enrollment, (b) Medicaid and Child Health Plus eligible, (c) primary caregiver age of 18 years or older, (d) sufficient understanding of English to complete study procedures and measures; exclusion criteria were (a) major medical problems and (b) evidence of dental caries at enrollment. Children on antibiotics were not excluded but a study visit was (re-)scheduled to occur at least 30 days from end of the antibiotic exposure (for valid collection of microbiology data). The study was approved by the local Institutional Review Board; written informed consent was obtained from a primary caregiver of participating children; participants were reimbursed for study participation.

### Procedure and measures

Children and a primary caregiver were seen at a university-based pediatric community clinic at enrollment and then at 6-month intervals for 2 years (i.e., up to 5 visits in total). Each visit consisted of behavioral assessments, parent-completed questionnaires, saliva collection, and a formal dental exam; each visit lasted approximately 2 h.

Caries status was assessed using the International Caries Detection and Assessment System (ICDAS) (12). At each visit, children were examined by a calibrated pediatric dentist (DKK) using standard protocol and procedures (12); all children had an ICDAS score of 0 at enrollment.

Oral microbiology was assessed from a sample of approximately 2 mL of saliva, which was collected prior to the dental exam at each visit. Microbiological profiles included *mutans streptococci*, *Lactobacilli* (LB), *Candida* species using established microbiological procedures (13). Whole, stimulated, saliva samples were obtained through a disposable saliva ejector attached to a 50 mL sterile centrifuge tube, which in turn was attached to a vacuum pump (14). All salivary samples were processed the same day using an Autoplate Spiral Plating System (Advanced Instruments, Inc.). Saliva samples were evaluated for MS, LB, and Candida species using Mitis Salivarius agar plus bacitracin, Rogosa, and CHROMagar respectively. For dispersion of cell clumps and dechaining of streptococci, a 2 mL aliquot of saliva was sonicated (three 10-s sonic bursts, 100 Watts of peak power). The suspension was serially diluted (10-fold dilutions) in phosphate buffer and 50 µL aliquots uniformly plated on: (i) Mitis Salivarius agar (Becton- Dickinson) supplemented with 20% sucrose and bacitracin (0.2 U/mL), to determine the presence of MS, (ii) Rogosa agar (Oxoid) to evaluate the presence of *Lactobacilli*, (iii) CHROMagar (BBL) supplemented with 0.1 mg/mL chloramphenicol to determine the presence of *Candida* spp., and (iv) tryptic soy agar supplemented with 5% sheep blood to enumerate the total microflora. MS plates were incubated at 37C in a 5% CO_2_ atmosphere, Rogosa plates were incubated anaerobically at 37C, CHROMagar plates were incubated aerobically at 37C and duplicated blood agar plates incubated at 37C both under aerobic and anaerobic conditions. All dilutions were plated in triplicates and the plates incubated under the described conditions for 72 h before colonies were counted. The number of *mutans streptococci*, *Lactobacilli*, *Candida* and total microflora were expressed as colony forming units (CFUs) per mL of saliva. Detailed procedures for collection, storage and analysis of oral microflora are well-established and described elsewhere (14). Salivary samples were processed between 1 and 4 h after collection, as there is no significant loss of numbers of total viable flora during the first 24 h (14).

Child and family stress exposures were based on parent-reported measures at each visit from six widely-used inventories assessing several types of child stress exposures: caregiver depression based on the Center for Epidemiologic Studies Depression Scale (15); anxiety and worry based on the Penn State Worry Questionnaire (16); alcohol use from the Alcohol Use Disorders Identification Test (17); stressful life events from a list of standard high-stress conditions [e.g., losses of income, health problems (18)]; household disorganization and confusion was derived from the Confusion, Hubbub, and Order Scale (19); violence exposure was based on the psychological aggression and physical assault subscales of the Conflict Tactics Scale (20) (following guidelines, this scale was administered yearly rather than at 6-monthly intervals). Following decades of clinical research practice in psychosocial studies (21) and current practice in clinical health studies, e.g., (22), a composite measure from standardized scores of each of the six scales was created at each visit because of the moderate-high correlations between scales, internal consistencies of a composite at each time point, and stability of each measure over the 2-year assessment period (see Supplementary Tables S1, S2).

Oral health behaviors and oral hygiene were assessed using a validated parent questionnaire (23) that includes questions related to the child's eating and drinking habits, snacking choices, sippy cup use, and the type and the amount of beverages and snacks consumed by the children; brushing behaviors; and the children's oral hygiene regimens. Items were combined into three subscales assessing Diet/Feeding (e.g., sippy cup use, having snacks during the day), Oral Hygiene (using fluoridated toothpaste, frequency of brushing), and Tooth Monitoring (e.g., whether or not the child has seen a dentist). These subscales at each assessment are considered as separate variables in the analytic models (see Supplementary Table S1).

Socio-demographic variables. Socio-demographic variables included the child's and parents/primary caregivers' age, race, ethnicity and gender; insurance status; parent/primary caregiver educational attainment and occupation; and income.

### Data analysis

Descriptive data and attrition analysis across the two-year assessment period are presented first. The primary psychosocial stress exposure variable is the composite psychosocial measure created by standardizing and summing each of the six measures; this was conducted at each assessment. The three measures of oral microbiology, *S. mutans*, *Candida*, and *Lactobacilli*, were log-transformed prior to analyses, following current practice. Prediction analyses were based on generalized estimating equations (GEE), which employs a regression model framework that accommodates repeated measurement within subject. The psychosocial stress composite was included as a time-varying covariate; several socio-demographic covariates, assessed from baseline (i.e., there was no or little variation in these factors over time), were included on an *a priori* basis: child sex, age, race/ethnicity, education, insurance status; other factors were included as covariates if there was reliable evidence of their association with exposure or outcome variables. Results from unadjusted and adjusted analyses of the association between psychosocial risk status and ECC are reported, followed by results from alternative models: psychosocial risk is included alongside (a) oral health behavioral variables to test the hypothesis that psychosocial risk associates with ECC because of its impact on oral hygiene; (b) oral microbiology to test the hypothesis that psychosocial stress exposure is associated with ECC onset via a link with microbiological risk for caries. The degree to which exposures in models (a) and (b) explained the psychosocial stress effect may be derived from comparing the estimate of psychosocial risk in the minimally adjusted model with the psychosocial risk estimate in alternative models described above. There is a lack of directly applicable prior research for the current analyses, e.g., for *a priori* estimating sample sizes needed to detect a psychosocial prediction of ECC onset. Instead, sample size justification for the current study was based on prior studies of conversion rates in young children (24).

## Results

### Descriptive and preliminary analyses

A total of 189 confirmed caries-free pre-school children was enrolled. Sample socio-demographic characteristics are displayed in Table 1, which indicate that the participating families are ethnically and racially diverse and at relatively high psychosocial and socio-demographic risk, e.g., based on parental educational attainment and Medicaid eligibility status and clinical measures (e.g., ≥20% above cut-offs scores for clinical measures). Of the *n* = 189 participants at baseline, retention was 72% from initial visit to 1 year and 95% from year 1 to year 2. There was no reliable evidence that demographic, oral hygiene or psychosocial factors in Table 1 predicted retention through the 2-year study period. The Appendix (Supplementary Table S1) provides descriptive data on predictors across the two-year study period. In general, there was minimal evidence of a linear increase or decrease in the study variables over the 2-year study period; stability estimates based on the Intra Class Correlation (ICC) varied somewhat across construct; oral microbiology markers were modestly/moderately stable. Preliminary data also indicated that, after accounting for child age, gender, race, and ethnicity, there was no additional prediction of ECC from other socio-demographic factors; as a result, prediction models include only child age, gender, race, and ethnicity as covariates.

**Table 1:** Social and demographic characteristics of the sample at enrollment.

	Mean (SD)/*n* (%)
Maternal education	
<High School	17 (9)
High School or GED	105 (55.5)
>High School	62 (32.8)
Marital/cohabiting status	
Single	104 (55)
Married/cohabiting	85 (45)
Employed	108 (57.1)
Smoking in the household (yes)	38/(20)
Number of people/house	4.1 (1.5)
Child sex (female)	91 (48.2)
Child age (months)	29.5 (9.1)
Child race	
Black/African-American	76 (40.2)
White	35 (18.5)
Mixed/other	78 (41.3)
Child ethnicity (Hispanic)	50 (26.5)
Child dental insurance	
Medicaid	143 (75.7)
Child health plus	35 (18.5)
Private	10 (5.3)
None	1 (.5)

Not all *n*'s sum to 189 because of missing response.

### Associations between psychosocial risk composite and oral health outcomes

Bivariate associations from GEE models indicated that the psychosocial risk composite was associated with select measures of oral health behaviors and microbiology (Table 2), in particular Diet/feeding [.92 (95% CI .22–1.61), *p* < .01], Oral hygiene [.39 (95% CI .09–.68)], *p* < .05), and higher concentrations *Lactobacilli* [.96 (.43–1.49), *p* < .001]. The psychosocial risk composite also predicted ECC onset over the course of the study (1.57 (95% CI 1.12–2.20, *p* < .001). The magnitude of the bivariate prediction of ECC onset from the psychosocial composite, oral health behaviors, and oral microbiology are provided in Supplementary Table S2.

**Table 2:** Bivariate associations between psychosocial risk composite and oral health behavior, oral microbiology, and ECC onset across the study period (based on generalized estimating equations).

	Estimate (SE)	95% CI	*p*
Oral health behavior			
Diet feeding	.92 (.35)	.22–1.61	.0098
Oral hygiene	.39 (.15)	.09–.68	.0102
Tooth monitoring	.03 (.06)	−.09–.14	.6543
Oral microbiology			
*S. Mutans*	.25 (.49)	−.71–1.21	.6117
*Lactobacilli*	.96 (.27)	.43–1.49	.0004
*Candida*	−.31 (.21)	−.73–.10	.1391
Early child caries onset	1.57 (.27)	1.12–2.20	.009

Early childhood caries is coded binary (1 = present, 0 = absent).

Table 3 presents Odds Ratio results from alternative GEE prediction models that examine ECC onset over the course of the study; for each model ECC onset is the outcome variable and child age, sex, and race and ethnicity were included as covariates. Model 1 in Table 3 indicates that psychosocial risk status is reliably associated with ECC status after accounting for covariates (OR 1.80, 95% CI 1.20–2.70, *p* < .01). Models 2 and 3 tested the hypothesis that the prediction from the psychosocial risk composite occurred via behavioral/oral health (Model 2) or via oral microbiology (Model 3). There was indirect support for the hypothesis that psychosocial risk exposure predicts ECC onset via behavioral and oral hygiene. Specifically, the prediction from the psychosocial composite was non-significant at *p* < .05 once parent-reported behavioral and oral hygiene factors were considered (from OR 1.80–1.53); the change in the psychosocial exposure estimate was modestly decreased. Additionally, none of the behavioral and oral hygiene measures was reliably associated with ECC with the psychosocial composite included.

**Table 3:** Prediction models of ECC onset: odds ratios from alternative GEE prediction models.

	Model 1 Estimate (SE) 95% CI	Model 2 Estimate (SE) 95% CI	Model 3 Estimate (SE) 95% CI
1. Psychosocial composite			
Psychosocial composite	1.80 (.37)**		
	1.20–2.70		
2. Psychosocial composite + oral hygiene			
Psychosocial composite		1.53 (0.41)	
		0.91–2.58	
Diet/Feeding		0.94 (0.04)	
		0.87–1.02	
Oral Hygiene		1.04 (0.08)	
		0.90–1.21	
Tooth Monitoring		1.29 (0.35)	
		0.70–2.19	
3. Psychosocial composite + oral microbiology			
Psychosocial composite			1.49 (0.39)
			0.89–2.50
*S. Mutans*			1.09 (0.04)*
			1.00–1.18
*Lactobacilli*			1.10 (0.06)+
			0.99–1.212
*Candida*			1.12 (0.06)*
			1.00–1.25
Model prediction accuracy:	58%	59.5%	69.1%

The outcome is the dichotomous measure of ECC onset, where 0 = ECC free and 1 = ECC onset. All models adjust for child age, sex, and race/ethnicity and time. Model 2 also includes Diet/Feeding, Oral Hygiene, Tooth Monitoring measures; Model 3 includes *S. Mutans, Lactobacilli, Candida*. Model prediction accuracy provides a measure of correct classification of ECC onset for models 1, 2, and 3.

** *p* < .01; * *p* < .05.

A different pattern of results was found in the model that included microbiology. As shown in Model 3 in Table 3, *S. mutans*, *Lactobacilli*, and *Candida* all significantly predicted ECC onset (*Lactobacilii* at *p* < .06) in the model that also included the psychosocial risk composite and covariates; as with Model 2, there was a modest decrease in the psychosocial composite prediction. The implication is that the prediction of oral microbiology to ECC operates separately from psychosocial risk.

### Supplementary analyses

Several sets of exploratory supplementary analyses were carried out. Additional supplementary analyses categorized the psychosocial composite according to >1 SD above the mean to assess if the findings were different using a more extremes approach to risk exposure; however, results were comparable to the continuous composite rating for oral health behaviors, oral microbiology, and caries onset. Data were also re-analyzed to examine sex differences in risk and health outcomes: models in Table 3 including child sex moderation indicated no reliable evidence that the prediction of ECC from psychosocial risk and covariates differed for boys and girls. Finally, supplementary analyses indicated that there was no individual psychosocial factor that was uniquely associated with ECC and other oral health outcomes: significant associations, for both bivariate and multivariable analyses, were found only with the composite measure that assessed risk across multiple exposures and not when individual factors were considered independently.

## Discussion

A long history of research has sought to determine if there are socio-demographic, psychosocial, or behavioral origins of childhood caries (25), in part, to complement (or confound) an oral microbiological model. Indirect evidence for a psychosocial explanation derives from the oral intervention research which repeatedly demonstrates only modest improvements in children's oral health outcomes, e.g., associated with varnish or antibiotics (26, 27). Stronger, but still quite indirect, support derives from many studies showing a socio-economic gradient of ECC onset. Findings from these sets of studies are less clear on two key questions addressed in the current study: is there evidence that *potentially modifiable psychosocial risks exposures* are reliably associated with ECC onset; second, if there is a reliable psychosocial risk prediction, then what is a plausible mechanism of effect? As discussed below, the current study provides firm affirmative evidence for the former and modest evidence regarding the second question.

Associations between specific, measured psychosocial factors and ECC onset has been widely reported (28, 29), including from diverse settings and populations (11, 30, 31), but interpretation of a putative causal role has been pre-empted by research design limitations, such as the reliance on cross-sectional data. In that regard, the 6-monthly assessment of *initially caries-free children* for two years provides important leverage for inferring that psychosocial risk led to subsequent ECC onset. Furthermore, finding a significant prediction after adjusting for socio-demographic covariates (i.e., Model 1 in Table 3), implies that the effects of psychosocial risk exposure on ECC can be distinguished from economic and demographic risk, which also predict ECC onset. Accordingly, the current findings offer considerable and novel support for a psychosocial stress-related account for caries onset in young children. Moreover, each of the factors included in the assessment of psychosocial risk has an established history in developmental and clinical research on children's behavioral health (32, 33), which has been extended more recently to physical health (34–37). Two additional points as regards clinical application warrant discussion. One is that the prediction derived from the psychosocial risk composite and not to any specific exposure in isolation. That is a consistent message in clinical research on child health, which has consistently emphasized the role of cumulative stress exposure across context, type, and time – and the corollary that targeting isolated markers will yield a weaker and a likely mis-specified effect. This observation does not necessarily impugn the possible benefits of targeted interventions (e.g., for parental depression), however, as interventions targeting any of the specific constructs included in the composite might be expected to have broader impact across a range of risks (e.g., parental depression-targeted interventions could be expected to have carry-over effects on family conflict). The second is that comparatively brief psychological-behavioral interventions such motivational interviewing (38) can be effective in reducing ECC, further suggesting that not all areas of risk exposure may need to targeted for an intervention to be effective – even if there is a complex set of risk exposures underlying the clinical condition.

If there is a reliable link between psychosocial risk exposure and ECC, then the subsequent question concerns how. Two leading possibilities were examined in this paper: via oral hygiene behaviors, for example, diet and brushing; or, via the oral microbiology that has a demonstrated causal role in ECC. In bivariate analyses the psychosocial risk composite was associated in several oral hygiene and oral microbiological risks (although not with *S. mutans*, which has the strongest biological claims on ECC). On the other hand, there was not clear evidence of either pathway from the regression models. That is, the psychosocial risk prediction was (only) modestly weaker in models that included a) oral hygiene or b) oral microbiological markers. The implication is that there is a modest confound between psychosocial risk and behavioral and oral hygiene factors in the prediction of ECC onset. On the other hand, the finding that each of the oral microbiological markers was a significant predictor of ECC onset when adjusting for psychosocial risk implies that these biological processes for ECC are not dependent on a psychosocial risk or stress biology marker associated with these risks.

The study has several limitations. First, the sample was selectively chosen to include very young children at greatest risk for ECC onset; the findings may not generalize to other populations. Similarly, the findings are based on a US sample; the findings obtained here may be particular to the health care context and patterns of oral health risks, which may differ widely across socio-cultural settings within and between countries, e.g., (39). Second, two of several possible mechanisms by which psychosocial risk may be associated with ECC onset were examined in detail. Further research is needed to replicate and integrate the current findings alongside alternative explanations concerning, for example, parental oral health and health care access, and the oral microbiome (40–42). Third, the follow-up period of two years was long enough to identify ECC onset in a sizable subsample, but likely the more severe cases; the current findings may not apply to later onset ECC. Finally, additional factors with potential relevance for ECC (e.g., sleep problems) were not systematically assessed or included in the analyses. Set against these limitations were several strengths of the paper, including a prospective assessment of initially caries-free children and extensive assessment of psychosocial risk exposure on multiple occasions.

There has been a major policy and clinical directive toward greater understanding of ECC and precision in specifying the mechanisms of ECC – in order to broaden and strengthen the types of intervention that may be needed. The current findings provide strong evidence that psychosocial risk is a contributor to ECC onset and add to a growing evidence base indicating a social and behavioral context that warrants attention in efforts to promote children's oral health.

## Data Availability

The raw data supporting the conclusions of this article will be made available by the authors, pending IRB approval.
