# Supplementary material for: Psychosocial determinants of oral health outcomes in young children
Source: Front Pediatr. 2024 Dec 6;12:1478302. doi: 10.3389/fped.2024.1478302 (PMC11659006; doi:10.3389/fped.2024.1478302)
Supplement: Supplementary file 1 [file Table1.docx]

| Appendix. Supplementary Table 1 | | | | | | | | | | | | | | | | |
| --- | --- | --- | --- | --- | --- | --- | --- | --- | --- | --- | --- | --- | --- | --- | --- | --- |
| **Variable** | **Visit** | | | | | | | | | | | | | | | **ICC** |
|  | 1 | | | 2 | | | 3 | | | 4 | | | 5 | | |  |
|  | N | Mean | Std Dev | N | Mean | Std Dev | N | Mean | Std Dev | N | Mean | Std Dev | N | Mean | Std Dev |  |
| AUDIT | 174 | 1.328 | 1.724 | 146 | 1.192 | 1.715 | 131 | 1.008 | 1.298 | 114 | 1.211 | 1.566 | 125 | 1.024 | 1.341 | 0.5000 |
| CES-D | 162 | 9.667 | 8.398 | 134 | 11.030 | 10.476 | 112 | 10.241 | 9.765 | 106 | 10.368 | 9.531 | 116 | 10.362 | 9.525 | 0.7400 |
| PSWQ | 172 | 43.738 | 14.517 | 137 | 43.255 | 14.781 | 126 | 42.833 | 15.075 | 106 | 43.123 | 15.712 | 115 | 43.130 | 15.451 | 0.8000 |
| CHAOS | 157 | 22.656 | 2.787 | 157 | 21.656 | 4.923 | 157 | 17.86 | 9.196 | 157 | 17.529 | 9.759 | 157 | 17.299 | 9.625 | 0.3629 |
| CTS | 155 | 41.935 | 25.253 | N/A |  |  | 143 | 39.664 | 27.711 | N/A |  |  | 153 | 38.484 | 28.506 | 0.3807 |
| SLE | 157 | 3 | 3.125 | 157 | 2.376 | 2.528 | 157 | 1.898 | 2.276 | 157 | 1.72 | 2.431 | 157 | 1.675 | 2.494 | 0.3093 |
| Diet_Feeding | 183 | 18.678 | 3.250 | 143 | 20.042 | 3.140 | 132 | 20.311 | 2.866 | 117 | 21.410 | 2.371 | 123 | 21.569 | 2.287 | 0.3884 |
| Oral_Hygiene | 183 | 7.678 | 1.647 | 149 | 8.040 | 1.899 | 131 | 7.817 | 1.616 | 119 | 7.840 | 2.042 | 127 | 7.906 | 2.166 | 0.5797 |
| Tooth_monitoring | 186 | 6.871 | 0.701 | 148 | 6.919 | 0.665 | 135 | 6.911 | 0.640 | 119 | 6.832 | 0.557 | 124 | 6.903 | 0.547 | 0.3296 |
| *S. Mutans* (log) | 182 | 6.170 | 5.078 | 150 | 6.126 | 4.813 | 135 | 6.356 | 4.812 | 120 | 6.629 | 4.495 | 130 | 6.898 | 4.635 | 0.6327 |
| *Lactobacilis* (log) | 179 | 0.728 | 2.085 | 150 | 1.143 | 2.638 | 135 | 1.182 | 2.564 | 120 | 1.497 | 2.910 | 130 | 1.668 | 3.220 | 0.2573 |
| *Candida* (log) | 179 | 1.162 | 2.625 | 150 | 1.164 | 2.509 | 135 | 0.975 | 2.067 | 120 | 0.805 | 1.938 | 130 | 1.153 | 2.721 | 0.5126 |

Note. AUDIT = Alcohol Use Disorders Identification Test; CES-D Center for Epidemiologic Studies Depression Scale; PSWQ = Penn State Worry Questionnaire; CHAOS = household disorganization and confusion scale; CTS = Conflict Tactics Scale; SLE = stressful life events (SLE). ICC = intraclass correlation coefficient.

Appendix. Supplementary Table 2.

Bivariate Associations between ECC onset and Psychosocial Composite, Oral Hygiene, and Oral Microbiological Markers.

ECC onset

Estimate (SE) 95% CI p

Psychosocial composite 1.57 (.27) 1.12 – 2.20 .009

Oral health behavior

Diet feeding 0.95 (0.04) 0.89--1.10 .107

Oral hygiene 1.01 (0.07) 0.88—1.16 .891

Tooth monitoring 1.16 (0.27) 0.73—1.83 .533

Oral microbiology

*S. Mutans* 1.13 (0.04) 1.05—1.22 0.001

*Lactobacilli* 1.14 (0.06) 1.03—1.27 0.010

*Candida* 1.16 (0.27) 0.73—1.83 0.533

______________________________________________________________________________

Note. Table shows the bivariate associations between each marker and ECC onset.
